# A Rare Case of Undifferentiated Pleomorphic Cardiac Sarcoma

**DOI:** 10.7759/cureus.59183

**Published:** 2024-04-28

**Authors:** Taulant Gishto, Leonard Simoni, Andi Kacani, Silvia Methoxha, Alessia Mehmeti

**Affiliations:** 1 Cardiovascular Disease, University Hospital Center "Mother Teresa", Tirana, ALB; 2 Cardiac Surgery, University Hospital Center "Mother Teresa", Tirana, ALB

**Keywords:** stable remission, systemic chemo therapy (stc), surgical excision of tumour, cardiac sarcoma, primary cardiac tumours

## Abstract

We present a case of a patient with a four-month history of gradual-onset dyspnea and generalized body weakness. During the clinical evaluation, a mass was found in the left atrium. Coronary angiography was performed and showed normal coronary arteries. We proceeded with a complete surgical excision of the tumoral mass, and histopathology confirmed it as undifferentiated cardiac sarcoma. Six months after the surgical intervention and adjuvant chemotherapy, the patient is in complete remission, with no evidence of a recurrence of the malignant pathology. Cardiac sarcoma is a rare clinical finding and a diagnostic and therapeutic challenge due to its numerous non-specific clinical presentations.

## Introduction

Primary cardiac sarcomas are rare malignant tumors that arise from the cardiac myocardium with a rapid local progression and a high rate of mortality, mainly through the infiltration of the myocardium by obstructing circulation or by distant metastases [1,2]. Diagnostic and management challenges arise due to the limitations of both biopsy acquisition and imaging-based diagnosis [3]. While complete surgical resection of the cardiac sarcoma is the optimal treatment, only in 12% of the cases is resection achieved for cure or complete remission (R0 resection), with a subsequent short median survival of typically six to twelve months. Salvage surgery, in most cases, is ineffective [1].

## Case presentation

**Figure 1:**
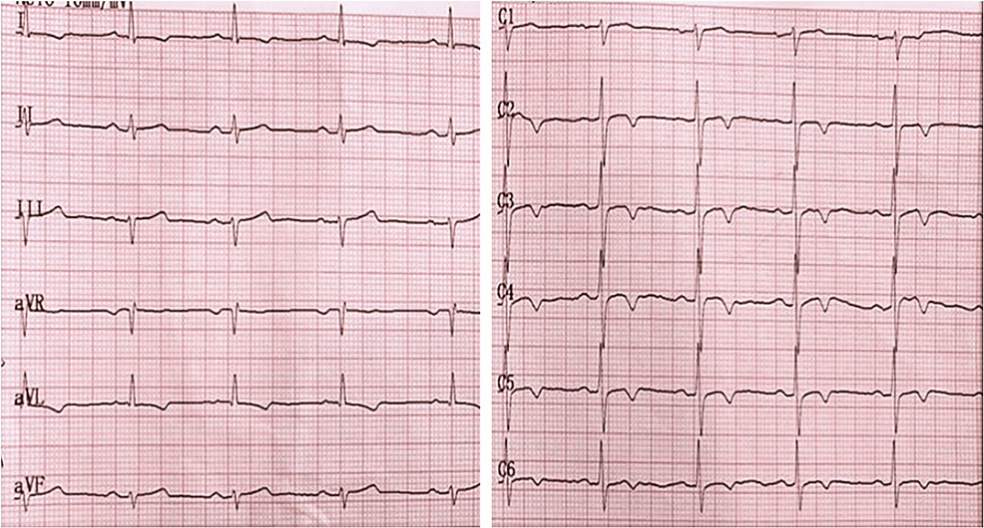
Admission ECG

A 72-year-old female with a history of hypertension and dyslipidemia presented to our cardiology clinic due to dyspnea with minimal exertion and rest, orthopnea, generalized weakness, and faintness. These symptoms had been progressively worsening for the past four months. On physical examination, the patient was alert, in good general condition, afebrile, and had normal vital signs. A systolic-diastolic murmur was evident at the apex during auscultation. Her resting ECG showed sinus rhythm, a heart rate of ~78 bpm, rS in D3, aVF, and negative T waves in aVL, V3-V6 (Figure 1). On admission, her complete blood count was altered, with hemoglobin levels of 8.8 g/dl, a hematocrit of 25.1%, and a red blood cell count of 298 K/μL; marked leukocytosis of 17600 K/μL; NTproBNP 1561 pg/mL (normal range <125 pg/mL); the rest of the biochemistry panel was within the normal range. Table 1 shows a summary of the laboratory results.

**Table 1:** Complete blood count and the biochemistry panel WBC: white blood cells, NEU: neutrophils, LYM: lymphocytes, RBC: red blood cells, HBA: hemoglobin, HCT: hematocrit, PLT: platelets, Creat: creatinine, Na: sodium, K: potassium, Cl: chloride, CRP: c reactive protein, Tot bilirubin: total bilirubin, AST: aspartate aminotransferase, ALT: alanine transaminase, CK: creatine kinase, CK-MB: creatine kinase-myoglobin binding, NTproBNP: N-terminal pro-b-type natriuretic peptide.

Parameters	Reference range	Units	Patient’s values
Complete blood count			
RBC	4–5.6	× 10^6^/μL	2.98
HCT	37–46	%	25.1
HB	12.1–15.9	g/dL	8.8
WBC	4–10.5	K/μL	17600
PLT	150–400	K/μL	323
Biochemistry panel			
Urea	21–43	mg/dL	28.7
Creatinine	0.57–1.11	mg/dL	0.63
Na	136–145	mmol/L	140
K	3.5–5.1	mmol/L	3.8
Cl	98–107	mmol/L	106
Tot bilirubin	0.3–1.2	mg/dL	0.56
ALT-SGPT	<55	U/L	10
AST-SGOT	5–34	U/L	19
CK	29–168	U/L	145
CK-MB	<3.1	ng/mL	1.0
Troponin-I	<0.016	ng/mL	0.010
NTproBNP	<125	pg/mL	1561.0
CRP	<0.5	mg/dL	0.47
Glucose	82–115	mg/dL	111

**Figure 2:**
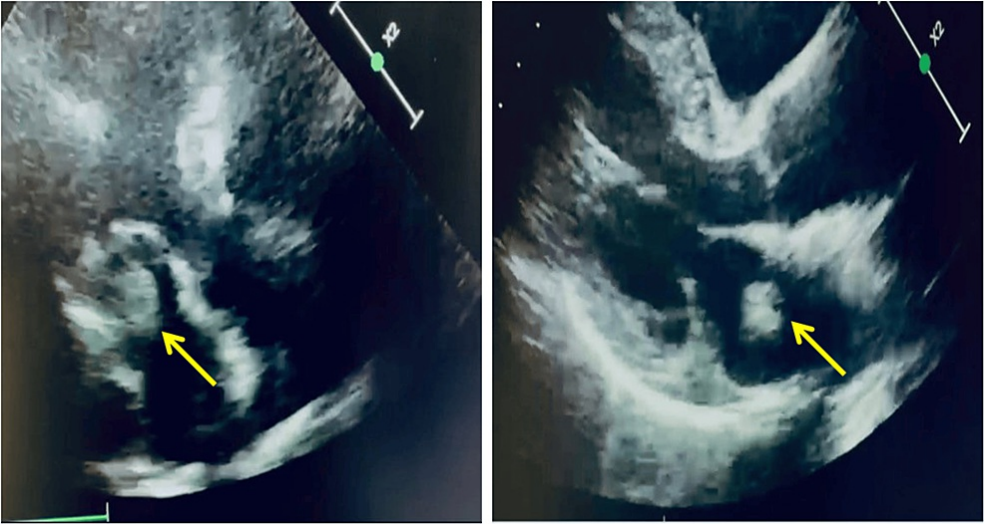
Transthoracic echocardiography of the cardiac mass pre-surgical intervention Arrow: the cardiac mass.

Transthoracic echocardiography (TTE) revealed a 27 mm × 25 mm formation in the left atrium, with little mobility. It is not clear whether it was also inserted on the posterior leaflet. The findings included a normal mitral valve gradient, dimensions of the left atrium, the opening and gradient of the aortic valve, and dimensions of the aortic root and ascending aorta. We also found the left ventricle with normal size, kinetics, and systolic function (Figure 2).

The surgical intervention was decided as the indicated treatment. Before the surgical procedure, the patient underwent coronary angiography, which ruled out the narrowing of the coronary arteries. The tumoral mass was suspected to be a cardiac myxoma, and due to the severe mitral obstruction and urgency of clinical symptoms, surgical intervention was performed directly on the day of admission.

**Figure 3:**
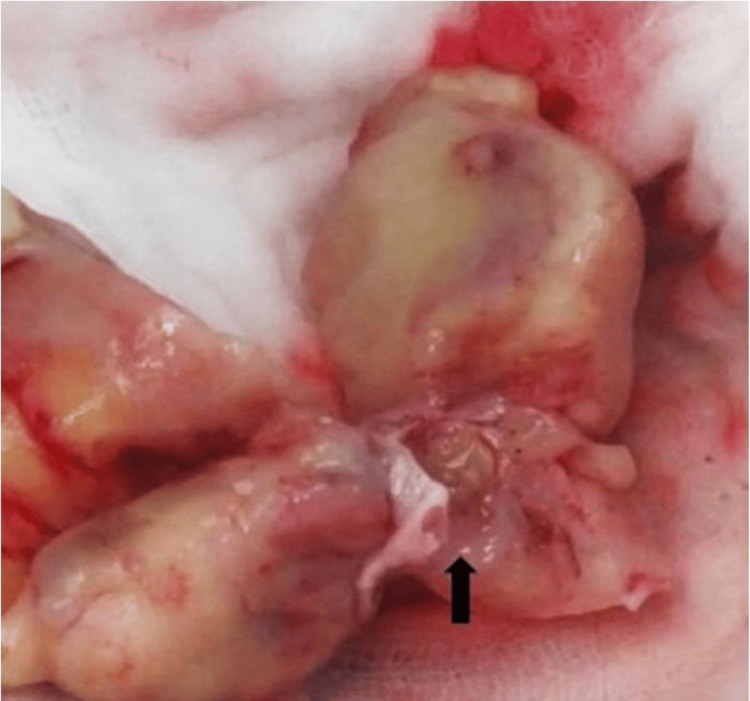
Macroscopic view of the mass after surgical excision Arrow: intraoperative mass.

**Figure 4:**
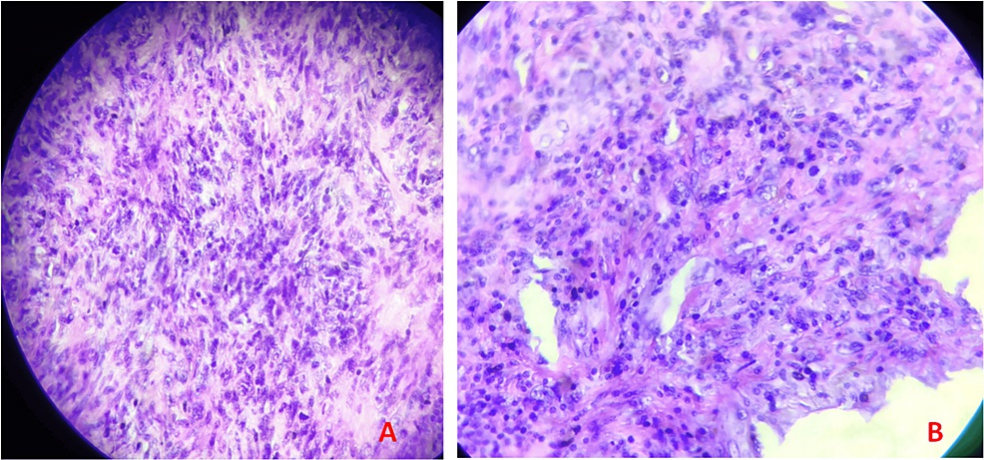
Histological examination: undifferentiated pleomorphic sarcoma (A) Small fascicles with marked cellularization with fusicellular cells on a fibro-inflammatory stroma and atypia is observed; (B) marked atypia and atypical mitosis.

Intraoperatively, a solid white mass was found attached to the posterior wall of the left atrium, infiltrating the anterior and posterior mitral leaflets, accompanied by retraction of the mitral leaflets. The tumor mass in the left atrium was excised, avoiding the mitral leaflets' perforation and allowing the leaflets' motility (Figure 3). The procedure was completed successfully and without complications. The material was taken for biopsy. Histopathology confirmed the tumor mass as undifferentiated pleomorphic sarcoma (Figure 4).

The patient was extubated 12 hours after the surgical intervention, facing a difficult recovery in the intensive care unit (ICU) due to respiratory failure. Recheck transthoracic cardiac echocardiography showed minimal signs of mitral regurgitation with no evidence of gross residual mass. The patient was discharged 16 days after the intervention in an improved condition.

**Figure 5:**
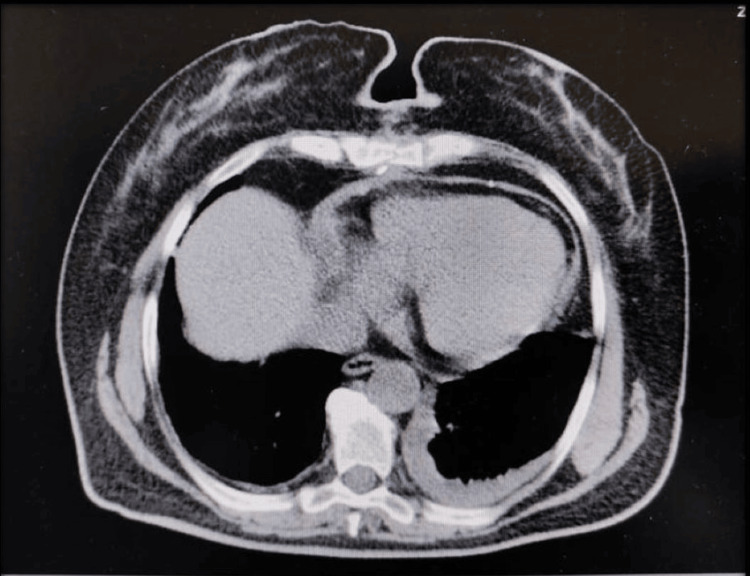
CT-scan performed after the surgical intervention, showing no residual gross mass, and minimal pericardial and pleural fluid

A total body CT scan was performed after the surgical intervention, revealing no evidence of a residual mass or distal metastasis, minimal pleural fluid, or minimal pericardial fluid. The CT scan revealed a 1.52 cm precarineal lymph nodule and a 1.42 cm paratracheal lymph nodule. All the other findings were normal (Figure 5).

The patient was discharged 16 days after the intervention in an improved condition. According to the treatment plan after surgery, the patient underwent systemic chemotherapy of six cycles of doxorubicin (75 mg/m^2^ per cycle). In a follow-up visit six months after the intervention, with normal vital parameters and an unremarkable physical examination, the transthoracic echocardiography of the control demonstrated a left ventricle with normal size, segmental kinetics, and systolic function (EF~0.68). Minimal mitral regurgitation was found.

## Discussion

According to the World Health Organization (WHO), the classification of cardiac neoplasms includes benign tumors, tumor-like lesions, malignant tumors, and pericardial tumors [4]. Cardiac tumors are divided into primary and secondary tumors, with a secondary/primary tumor ratio of 20:1 [4]. About 10% of primary cardiac tumors are malignant, while 90% are benign, mainly myxoma [5].

Cardiac sarcomas account for about 1% of all soft tissue sarcomas and are the most common form of cardiac malignancy, with the average age of presentation ranging from 39 to 44 years. Most sarcomas (histiocytomas, malignant fibrosis, leiomyosarcomas, myxoid sarcomas, osteosarcomas, and undifferentiated sarcomas) originate from the left atrium [6].

Three main mechanisms cause cardiac tumor symptoms: obstruction, embolization, and arrhythmia. Very rarely, pericardial effusion and tamponade may be the initial manifestations of the disease. Both atrial and ventricular tumors can cause obstructive symptoms manifested as syncope, chest pain, dyspnea, or heart failure. The finding of a cardiac mass accompanied by pericardial effusion should raise the suspicion of a malignant cardiac tumor [7]. Twenty-nine percent of cardiac sarcomas are associated with metastases at the presentation time, mainly in the lungs [8].

Radiographic, laboratory, and electrocardiographic findings are nonspecific. The first diagnostic approach is two-dimensional cardiac echocardiography, due to its widespread availability. Meanwhile, transesophageal echocardiography may be used as a diagnostic modality in certain cases, such as in patients who are overweight or have chronic obstructive pulmonary disease. Cardiac CT offers clear visualization of intra-cardiac masses and can assess the extent of myocardial and pericardial involvement. Cardiac MRI offers a wider field of view, no radiation exposure, and detailed tumor characterization with functional assessment. This combination enables a more accurate diagnosis and treatment plan [9]. The evaluation of a cardiac tumor aims to inform us about the size, morphology, anatomical location, attachment site, mobility, and relationship to the cardiac valves and chambers, vascular supply, pericardial effusion presence, pulmonary or vena cava obstruction, and extracardiac findings [5].

In most cases, undifferentiated sarcomas are typically located in the left atrium, presenting as a discrete mass and as an infiltrative and irregular mass accompanied by necrosis and hemorrhage. Cardiac FSE images reveal an isointense contrast between the tumor and the myocardium [9].

Despite advancements in non-invasive imaging modalities, which are of great value in the characterization of the mass, histopathological examination is required to establish a definitive diagnosis [10].

Complete surgical resection of cardiac sarcomas remains the optimal treatment based on studies showing that R0 resection improves survival compared with incomplete resection. Total resection must be balanced with cardiac function preservation for better intervention outcomes (lower mortality rate) [11]. Postoperative therapy (chemotherapy, radiotherapy, or a combination of both) is associated with better average survival after surgical resection of the cardiac tumor [12]. The most commonly used chemotherapeutic regimen for cardiac sarcomas is the combination of ifosfamide and doxorubicin [8]. Cardiac sarcomas, unfortunately, have a poor prognosis, with an average survival of 6 months to 25 months after diagnosis [7,13].

## Conclusions

Cardiac sarcoma is a rare clinical finding and a diagnostic and therapeutic challenge due to its numerous non-specific clinical presentations. The role of diagnostic imaging, which guides us toward the presence of a cardiac tumor mass and its characteristics, should not be overlooked. Intraoperative findings and histopathology are used to confirm the diagnosis. Our reported case presented a successful outcome with no recurrence at six months post-surgery of an undifferentiated cardiac sarcoma, attributed to early clinical suspicion, guiding imaging, complete resection, and adjuvant chemotherapy. A multidisciplinary approach played a key role in achieving a positive outcome.
